# Supplementary figures and images for: RPG acts as a central determinant for infectosome formation and cellular polarization during intracellular rhizobial infections
Source: eLife. 2023 Mar 1;12:e80741. doi: 10.7554/eLife.80741 (PMC9991063; doi:10.7554/eLife.80741)

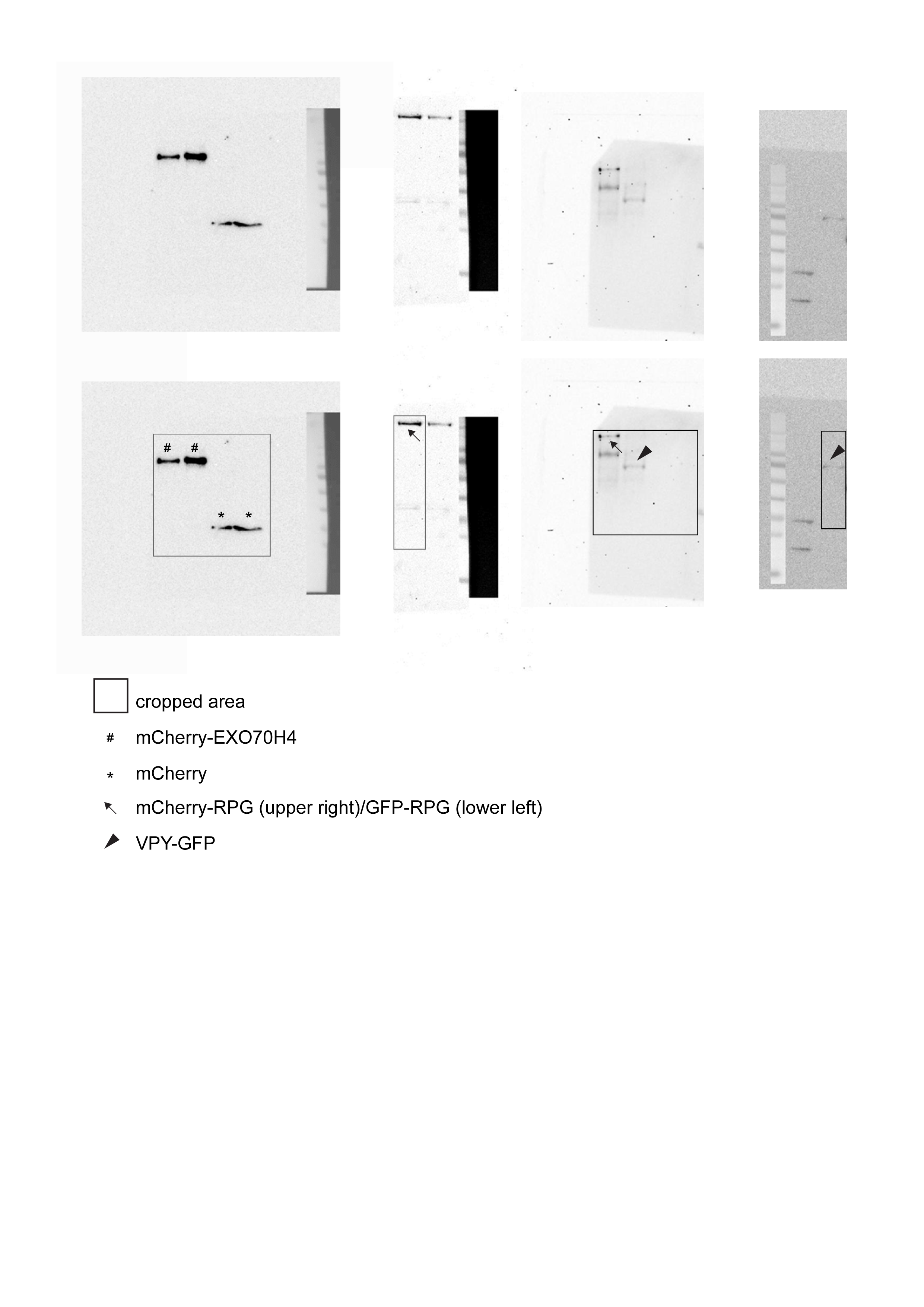

Supplement: Figure 3—figure supplement 7—source data 1. [file elife-80741-fig3-figsupp7-data1.zip › Figure 3 - figure supplement 7 - source data 1/Figure 3-figure supplement 7A - WB_with annotation.tif]

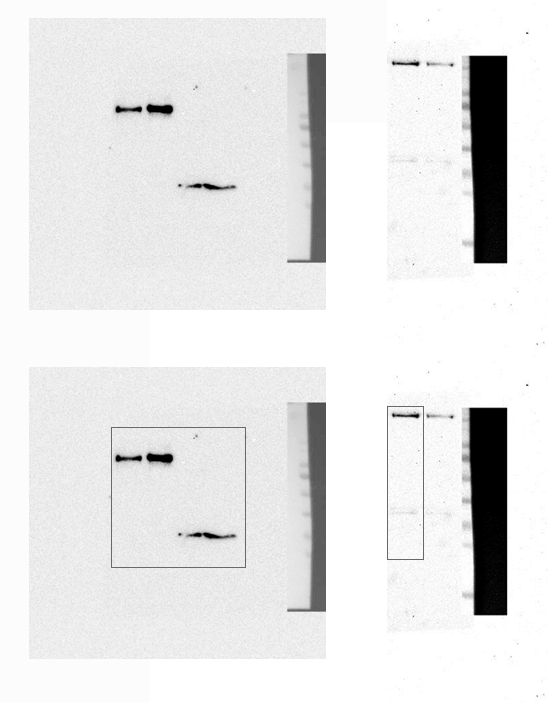

Supplement: Figure 3—figure supplement 7—source data 1. [file elife-80741-fig3-figsupp7-data1.zip › Figure 3 - figure supplement 7 - source data 1/Figure 3-figure supplement 7A_WB_anti-mCherry_raw.tif]

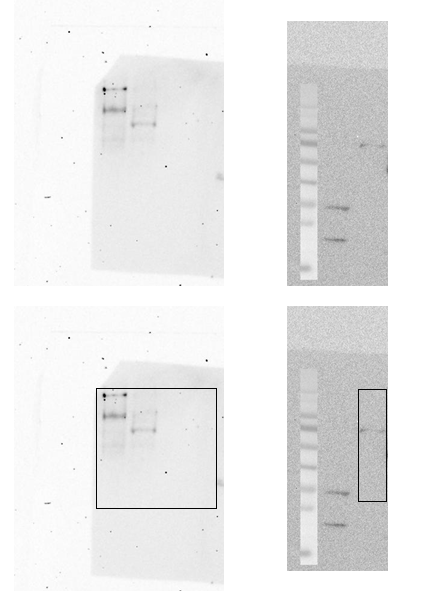

Supplement: Figure 3—figure supplement 7—source data 1. [file elife-80741-fig3-figsupp7-data1.zip › Figure 3 - figure supplement 7 - source data 1/Figure 3-figure supplement 7A_WB_anti-mGFP_raw.tif]

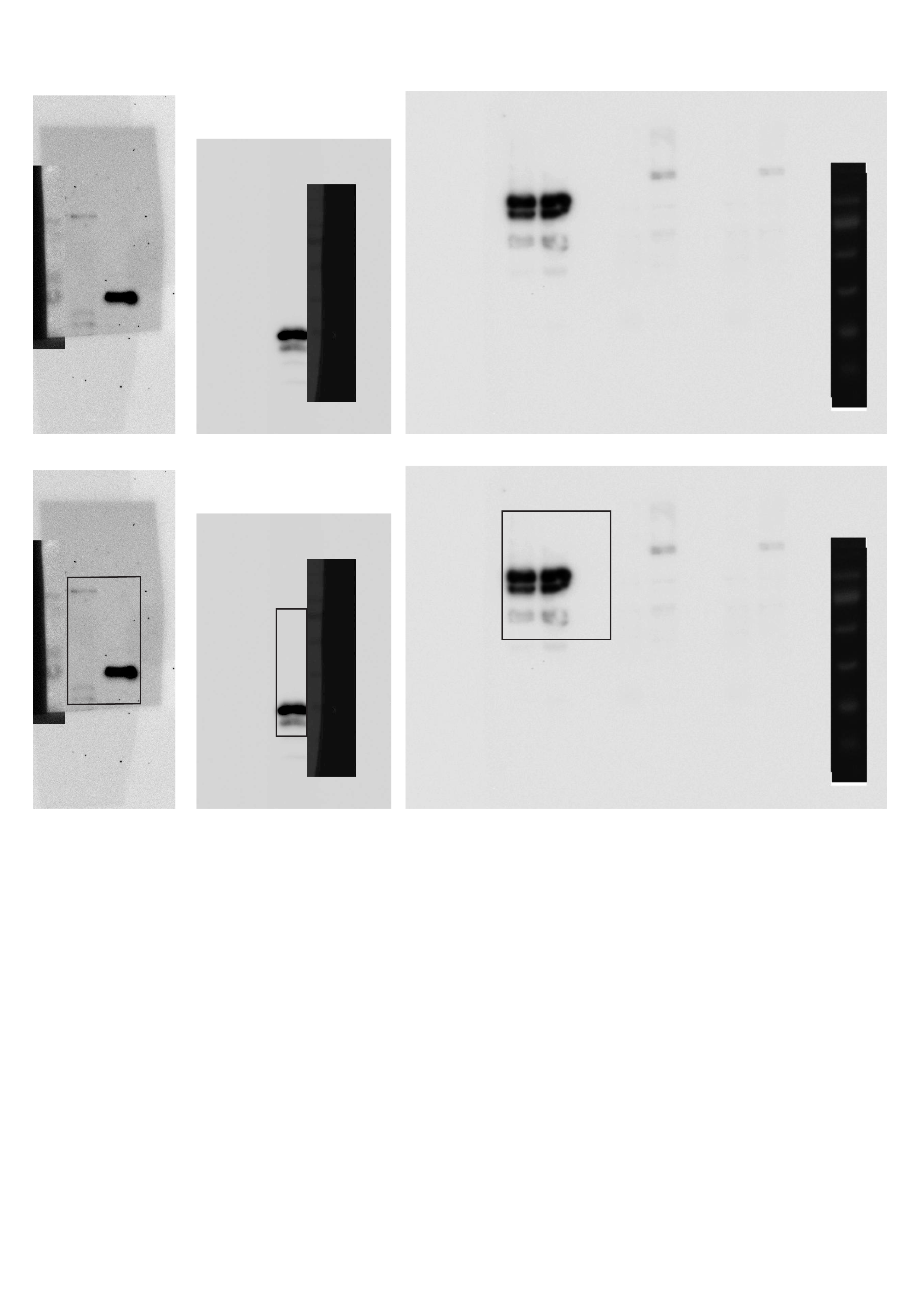

Supplement: Figure 3—figure supplement 7—source data 1. [file elife-80741-fig3-figsupp7-data1.zip › Figure 3 - figure supplement 7 - source data 1/Figure 3-figure supplement 7B WB raw.tif]

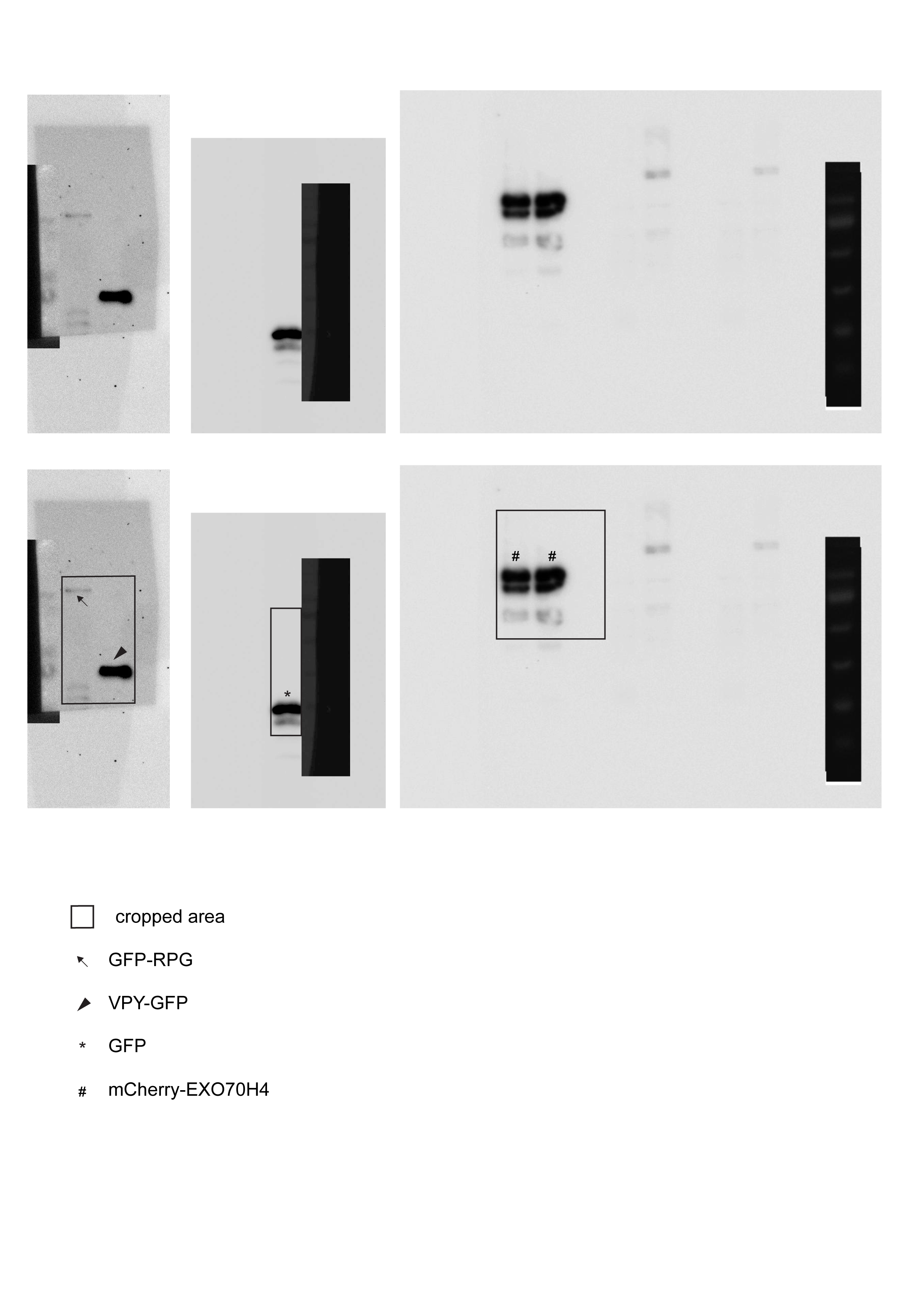

Supplement: Figure 3—figure supplement 7—source data 1. [file elife-80741-fig3-figsupp7-data1.zip › Figure 3 - figure supplement 7 - source data 1/Figure 3-figure supplement 7B WB with annotation.tif]

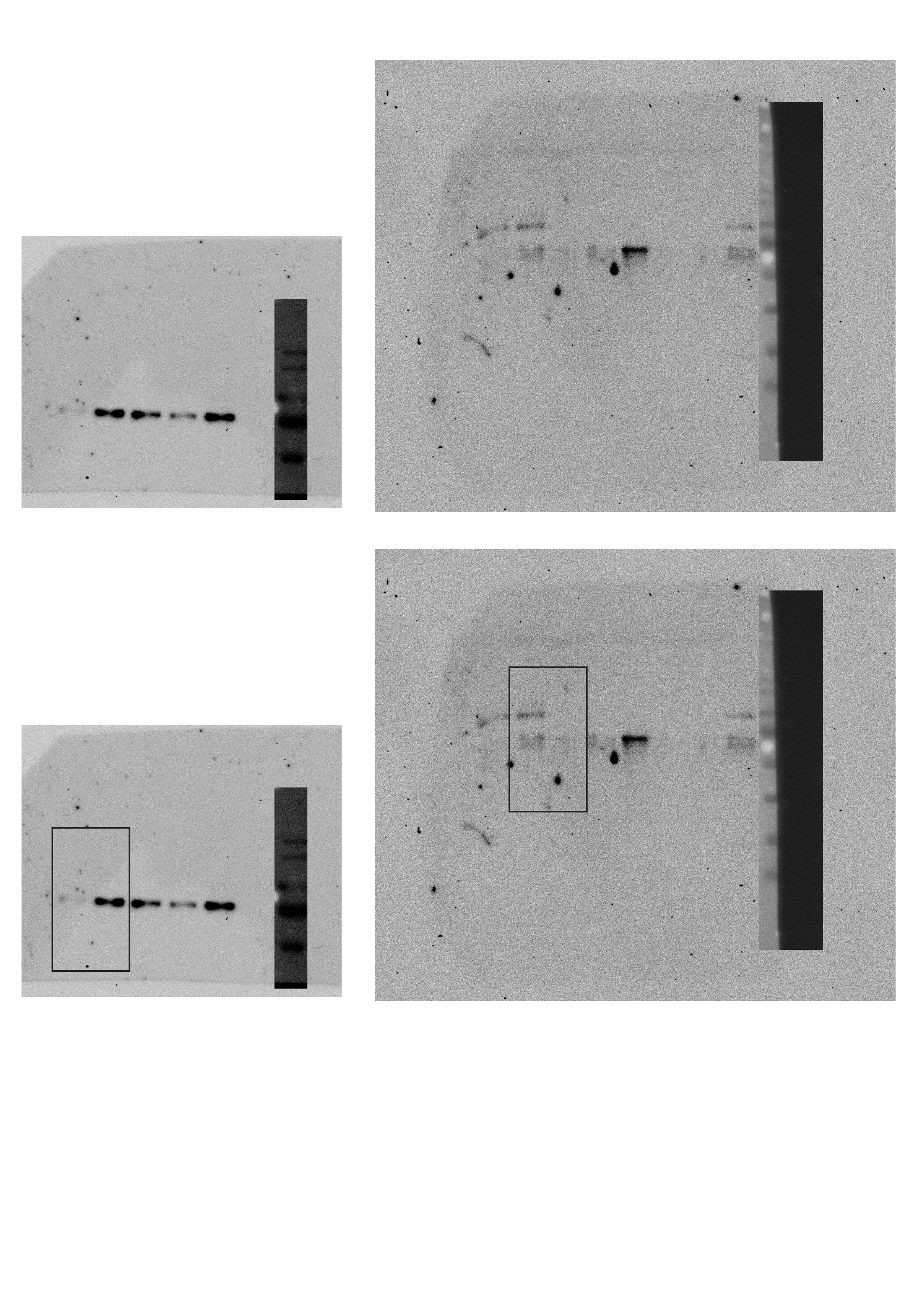

Supplement: Figure 3—figure supplement 7—source data 1. [file elife-80741-fig3-figsupp7-data1.zip › Figure 3 - figure supplement 7 - source data 1/Figure 3-figure supplement 7C WB raw.tif]

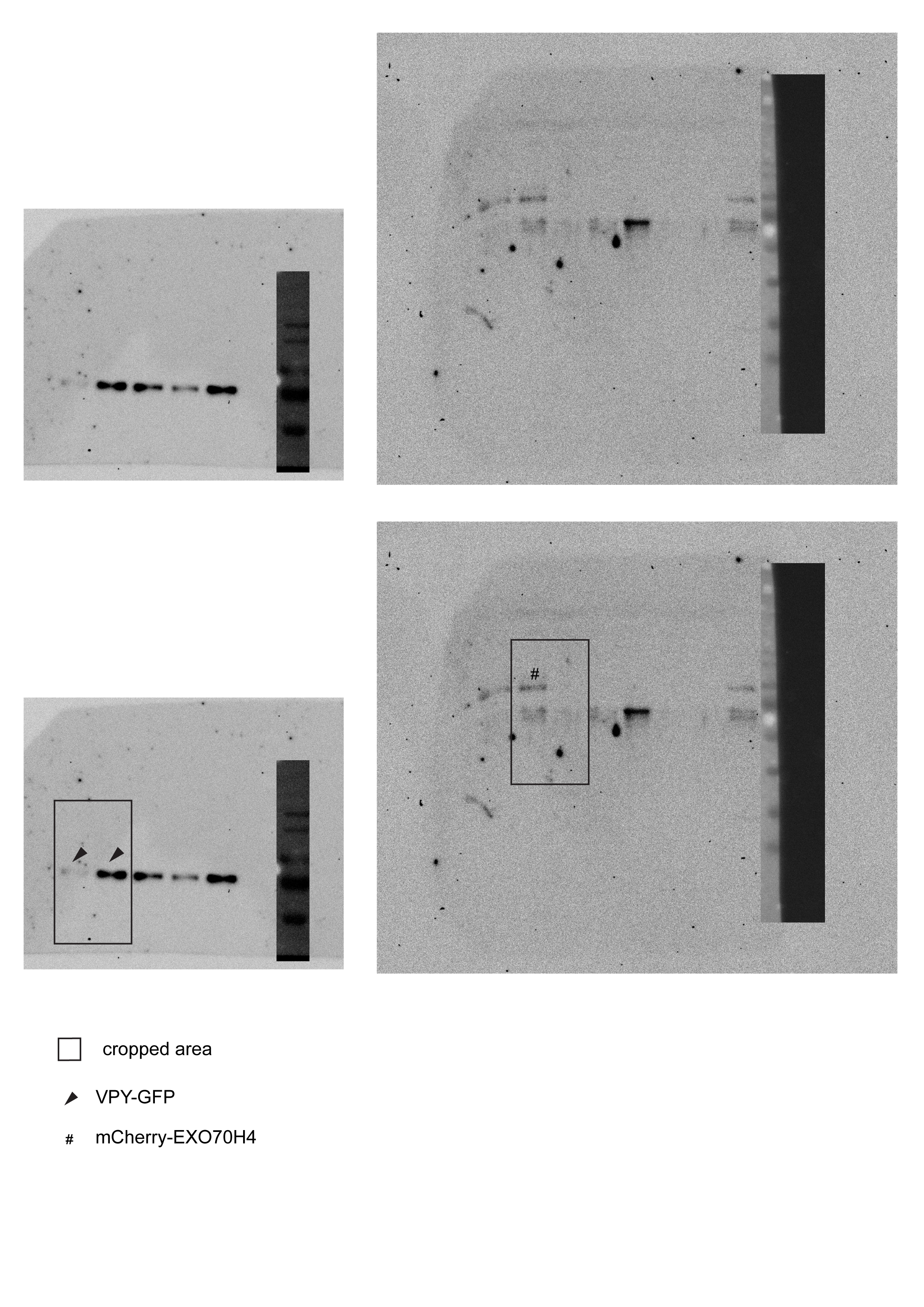

Supplement: Figure 3—figure supplement 7—source data 1. [file elife-80741-fig3-figsupp7-data1.zip › Figure 3 - figure supplement 7 - source data 1/Figure 3-figure supplement 7C WB with annotation.tif]
